# *Leishmania (Viannia) braziliensis* type 2 as probable etiological agent of canine cutaneous leishmaniasis in Brazilian Amazon

**DOI:** 10.1371/journal.pone.0216291

**Published:** 2019-04-30

**Authors:** Andreia Fernandes Brilhante, Luciana Lima, Ricardo Andrade Zampieri, Vânia Lúcia Brandão Nunes, Maria Elizabeth Cavalheiros Dorval, Patrícia Fernandes Nunes da Silva Malavazi, Leonardo Augusto Kohara Melchior, Edna Aoba Yassui Ishikawa, Cristiane de Oliveira Cardoso, Lucile Maria Floeter-Winter, Marta Maria Geraldes Teixeira, Eunice Aparecida Bianchi Galati

**Affiliations:** 1 Faculty of Public Health, University of São Paulo, São Paulo, Brazil; 2 Federal University of Acre, Rio Branco, Acre, Brazil; 3 Institute of Biomedical Sciences, University of São Paulo, São Paulo, Brazil; 4 Institute of Biosciences, University of São Paulo, São Paulo, Brazil; 5 Laboratory of Parasitology, Anhanguera-Uniderp University, Campo Grande, Mato Grosso do Sul, Brazil; 6 Laboratory of Clinical Analysis, Federal University of Mato Grosso do Sul, Campo Grande, Mato Grosso do Sul, Brazil; 7 Nucleus of Tropical Medicine, Federal University of Pará, Belém, Pará, Brazil; Academic Medical Centre, NETHERLANDS

## Abstract

Canine cutaneous leishmaniasis (CCL) is a zoonosis of public health interest, and in the Americas, *Leishmania* (*Viannia*) *braziliensis* has been identified as the main etiological agent. The present study sought to investigate *Leishmania* spp. infection in domestic dogs from a rural area of the Xapuri municipality, Acre state, Brazilian Amazonia. For this purpose, visits were carried out to domiciles where the human cases of American cutaneous leishmaniasis (ACL) occurred, followed by the clinical evaluation of the animals in search of clinical signs suggestive of CCL. Blood samples were collected from 40 dogs, 13 of which had lesions suggestive of CCL, and biopsies of these lesions were performed. The methods used were Neal, Novy, and Nicolle’s (NNN) medium cultures and direct parasitological examination. Further, to detect and characterize *Leishmania* DNA some molecular techniques were performed such as conventional polymerase chain reaction (PCR) and sequencing targeting SSU rDNA and ITS1, restriction fragment length polymorphism (RFLP) and high resolution melting (HRM) analysis targeting hsp70. The investigation revealed that the results obtained from the parasitological methods were negative. In PCR by ITS1 and network topology sequences, six strains from dogs, isolated from the Peruvian Andes, appeared identical to *Leishmania* (*Viannia*) *braziliensis* type 2 (99–100%). By other molecular methods these samples turned out to be positive to *Leishmania* (*Viannia*) sp.. The diagnosis of *Leishmania* in domestic dogs from Acre state showed a high proportion of infected animals, and the occurrence of *L*. *braziliensis* type 2 in Brazil for the first time. This new report suggests that *L*. *braziliensis* type 2 is both trans- and cis-Andean. However, more studies are needed regarding the clinical and diagnostic aspects of this species of *Leishmania*.

## Introduction

In Brazil, there are different etiological agents involved in the dermotropic forms of the disease, with *Leishmania* (*Viannia*) *braziliensis* and *Leishmania* (*Leishmania*) *amazonensis* presenting the greatest geographical distribution. The largest numbers of cases of the disease have been reported in the northern region which possesses the highest diversity of *Leishmania* species, in addition to reservoirs and proven or incriminated vectors [1, 2].

The domestic dog plays a significant role in the transmission cycle of *Leishmania* (*Leishmania*) *infantum*, the agent of American visceral leishmaniasis (AVL) in the Americas [3] and frequently canine cases precede the occurrence of the disease in humans [4]. However, the role of the dog in the transmission cycle of etiological agents of ACL is not well understood, whereas canine cases have generally been found in association with *L*. (*V*.) *braziliensis* in several regions of Brazil [5–7].

In recent years, the state of Acre has been considered to present one of the highest prevalences in the Brazilian Amazonian region and also in Brazil as a whole [8]. The municipality of Xapuri, where this study was carried out, is one of those with the highest reported number of cases and which contributes significantly to the increase of the prevalence of the disease in the state as a whole [9]. Exactly as with humans, domestic dogs can be affected by ACL. However, no studies have been undertaken in the region to evaluate the relationship of these animals to *Leishmania* spp. Therefore, the present report describes canine cases of ACL from the Xapuri municipality, Brazilian Amazon, attributed to *Leishmania* (*Viannia*) *braziliensis* type 2, a distinct species from *L*. (*V*.) *braziliensis* type 1 that has no documented clinical records, and has previously been found only in the Peruvian Andes.

## Methods

### Study area and sampling

The study was carried out in a rural, forested area in Xapuri municipality about 175 km from Rio Branco, the Acre state capital, where human and canine cases of ACL have been reported. The primitive vegetation of Xapuri is typical of the Amazonian biome, characterized by a tropical climate with abundant rainfall from October to April and a dry season from May to September. The average annual temperature is 27°C, and the human population consists of about 17,000 inhabitants. The local economy mainly depends on latex, Brazil nut extractivism [10, 11].

According to information obtained from the Xapuri Health Surveillance Office regarding the occurrence of human cases of ACL, visits to the patients’ homes were carried out between July and October 2014, in areas of the municipality, composed of small properties such as farms and forests used for rubber extraction.

After the owners’ authorization had been obtained, their dogs were clinically examined for the purpose of identifying manifestations suggestive of leishmaniasis. Approximately 5.0 ml of venous blood was collected by jugular or cephalic vein puncture and stored in plastic tubes with and without anticoagulant (ethylenediamine tetraacetic acid; EDTA) for molecular tests.

The animals that presented lesions suggestive of ACL were anaesthetized to collect fragments of the lesion in tubes containing absolute ethanol and antibiotic saline solution (Gentamicin). These fragments were submitted to parasitological and molecular techniques to confirm the infection and to identify *Leishmania* spp.

### Parasitological tests

The smears were prepared by apposition from the fragments of the lesions, which after fixation with methyl alcohol were stained with Giemsa and examined for amastigote forms of *Leishmania* spp. For the isolation of the parasite, parts of the fragments of the lesions were immersed in saline solution with gentamicin sulfate for 24 hours at 4°C, followed by the seeding of the blood samples in NNN culture medium. The cultures were kept in a BOD (Biochemical Oxygen Demand) incubator at 25°C and examined weekly for 60 days.

### Molecular assays

#### DNA extraction and *Leishmania* spp. detection

Blood samples and the fragments of the lesions were submitted to the DNA extraction protocol described by Adams *et al*. [12]. For the molecular diagnosis, various techniques and primers were utilized, as follows.

#### Nested-PCR SSU rRNA and PCR-RFLP hsp70C

Nested-PCR was performed on the samples of total blood and tissues of the animals, according to the technique described by Savani *et al*. [13], which amplifies a region of the SSU rRNA gene of trypanosomatids. Also were produced hsp70C fragments according to the description given by Graça *et al*. (2012) [14], using the pre-amplification products described in High Resolution Melting (HRM) analysis as a template.

#### Internal transcribed spacer 1 (ITS1)

This PCR was carried out targeting the internal transcribed spacer 1 (ITS1) using the primers LITSR—Forward (5′-CTG GAT CAT TTT CCG ATG-3′) and L5.8S - Reverse (5′-TGA TAC CAC TTA TCG CAC TT-3′) to detect the infection caused by *Leishmania* spp. The PCR conditions were fulfilled according to the details outlined by Schönian *et al*. (2003) [15]. PCR products obtained were cloned, followed by the sequencing of 2 to 4 clones from each isolate.

The ITS1 sequences were aligned using Clustal X [16] and manually refined. Network genealogy was inferred by SplitsTree v4.11.3 using the neighbor-net method [17]. Internode supports were estimated, by performing 100 bootstrap replicates using the parameters which were optimized for network inferences.

#### High resolution melting (HRM) analysis targeting hsp70

A pre-amplification PCR step was performed using primers hsp70-preamp-F: 5’-GGCATCCTGAACGTGTCCG-3’ and hsp70-preamp-R: 5’-ATCTTGGTCATGATCGGGTTGCAT-3’. Thousand-fold dilutions from the pre-amplification reactions were used as a template in HRM tests described by Zampieri *et al*. (2016) [18]. An additional target, called Amplicon 3 using the primer hsp70F3: 5’ GTCGACGCTGAACAAGGAGATCGA-3’ and hsp70C reverse, described by Graça *et al*. (2012), was used [14]. Genomic DNA samples from reference-strains of *L*. (*L*.) *infantum*, *L*. (*L*.) *amazonensis*, *L*. (*L*.) *mexicana*, *L*. (*V*.) *lainsoni*, *L*. (*V*.) *braziliensis*, *L*. (*V*.) *guyanensis*, *L*. (*V*.) *naiffi* and *L*. (*V*.) *shawi* were used as standards in all the HRM tests. All reactions were performed in a StepOne Real-Time PCR System, and data analysis was undertaken using High-Resolution Melt Software v3.0.1 (Thermo Fisher Scientific, Walthan, MA, USA).

### Ethical considerations

The biopsies and blood sampling from dogs were performed by professional veterinarians, respecting international recommendations for animal welfare, with the approval of the Ethics Committee for the Use of Animals for Experimentation of the Federal University of Acre (Comite de Ética no Uso de Animais para Experimentação da Universidade Federal do Acre–CEUA-UFAC) under opinion number 23107.019254 / 2013–31 and according to national law n°11794/2008 by the National Council for the Control of Animal Experimentation (Conselho Nacional de Controle de Experimentação Animal–CONCEA).

## Results

**Fig 1:**
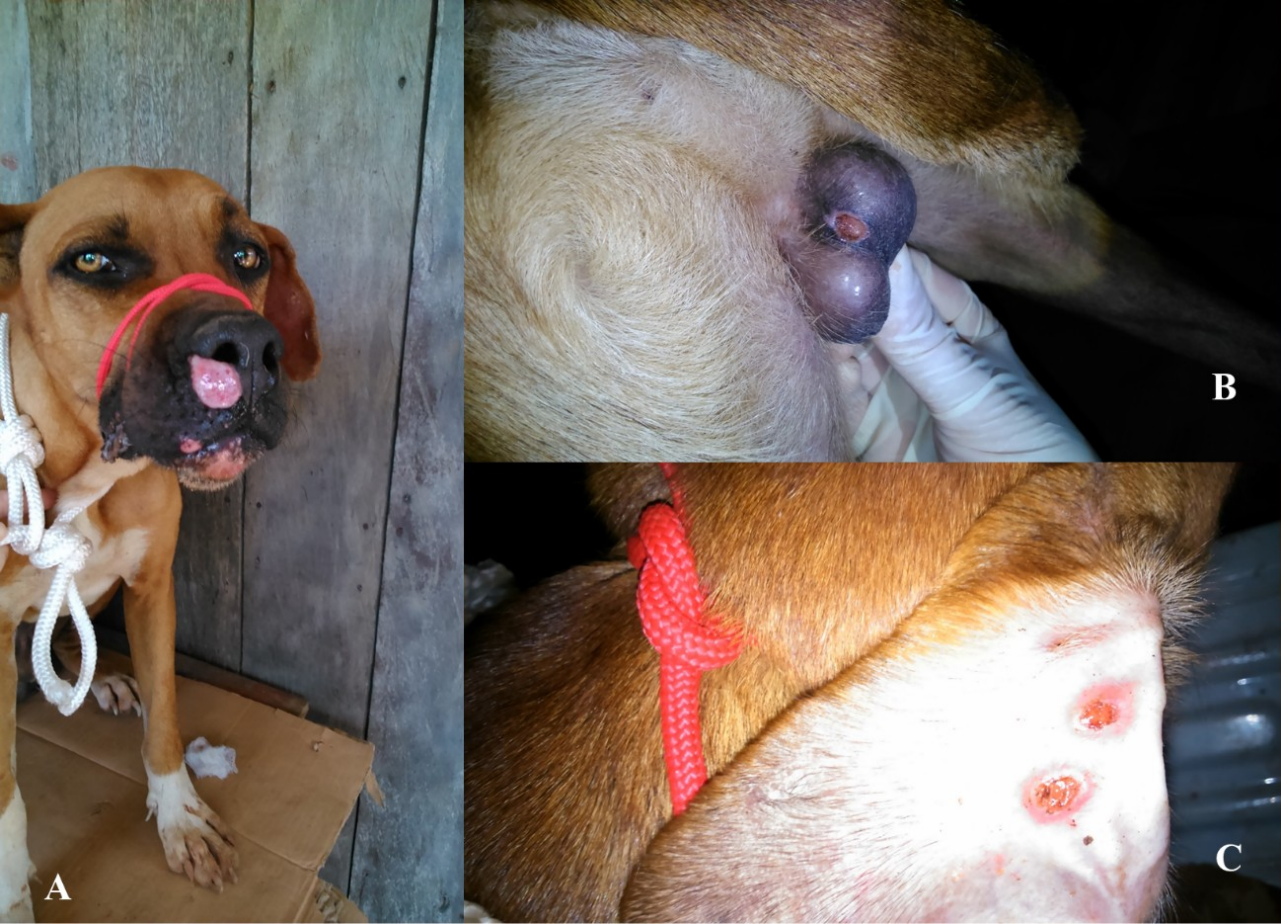
The figure presents the photographic registry of dogs with lesions. A: dog with the mucous lesion on the nose. B: dog with the scrotal lesion. C: dog presenting ulcerative lesions on the edge of the ear.

A total of 40 dogs (33 males and 7 females) were investigated, of which 13 presented lesions suggestive of ACL. In eight of them, the lesions were simple. In the other five there were lesions on the mucosa and/or muzzle. Three animals had cutaneous lesions in the scrotum, two of which also had mucosal lesions on the muzzle, and one animal had ulcerative lesions on the ear, muzzle and scrotum (Fig 1).

None of the cultures obtained from the fragments of the lesions and blood samples of these animals presented promastigote forms during the period of 60 days after sowing. The Giemsa slides obtained from the fragments of the lesions were negative.

Table 1 illustrates the result of the molecular analyses and the markers used. The blood samples were negative by all the molecular techniques. Moreover, of the 13 animals from which a biopsy of cutaneous lesion was obtained, eight showed the presence of *Leishmania* spp. with Nested-PCR S17/S18.

**Table 1:** Detection of *Leishmania* in biopsy lesion samples obtained from domestic dogs by the PCR and sequence analysis by SSU rRNA and ITS1, RFLP hsp70C and HRM analysis targeting hsp70.

Sample	SSU rRNA	ITS1	RFLP hsp70C	HRM
Dog 01 –Chorinho	*Leishmania* (*Viannia*) sp.	Negative	*Leishmania* (*Viannia*) *braziliensis*	*Leishmania* (*Viannia*) variants
Dog 01 –Bandeira	Negative	Negative	N/A	N/A
Dog 02 –Hulk	Negative	Negative	Negative	Negative
Dog 03 –Halley	Negative	*Leishmania* (*Viannia*) *braziliensis* type 2	*Leishmania* (*Viannia*) *braziliensis*	
Dog 05 –Help	*Leishmania* (*Viannia*) sp.	Negative	*Leishmania* (*Viannia*) *braziliensis*	*Leishmania* (*Viannia*) *braziliensis*
Dog 06 –Negão	*Leishmania* (*Viannia*) sp.	Negative	*Leishmania* (*Viannia*) *braziliensis*	*Leishmania* (*Viannia*) variants
Dog 11 –Valente	Negative	Negative	Negative	Negative
Dog 15 –LN–Xapuri	Negative	*Leishmania* (*Viannia*) *braziliensis* type 2	*Leishmania* (*Viannia*) *braziliensis*	*Leishmania* (*Viannia*) variants
Dog 15 –LP–Xapuri	Negative	Negative	Negative	Negative
Dog 26 –LN–Marmaduque	*Leishmania* (*Viannia*) sp.	*Leishmania* (*Viannia*) *braziliensis* type 2	*Leishmania* (*Viannia*) *braziliensis*	*Leishmania* (*Viannia*) variants
Dog 26 –LP–Marmaduque	*Leishmania* (*Viannia*) sp.	Negative	*Leishmania* (*Viannia*) *braziliensis*	*Leishmania* (*Viannia*) variants
Dog 37 –Xorinho 1	*Leishmania* (*Viannia*) sp.	*Leishmania* (*Viannia*) *braziliensis* type 2	*Leishmania* (*Viannia*) *braziliensis*	*Leishmania* (*Viannia*) variants
Dog 39 –Pantera	*Leishmania* (*Viannia*) sp.	Negative	*Leishmania* (*Viannia*) *braziliensis*	*Leishmania* (*Viannia*) variants
Dog 40 –Bethoven	*Leishmania* (*Viannia*) sp.	*Leishmania* (*Viannia*) *braziliensis* type 2	*Leishmania* (*Viannia*) *braziliensis*	*Leishmania* (*Viannia*) variants
Dog 41 –Xorinho 3	*Leishmania* (*Viannia*) sp.	*Leishmania* (*Viannia*) *braziliensis* type 2	*Leishmania* (*Viannia*) *braziliensis*	*Leishmania* (*Viannia*) variants

**Fig 2:**
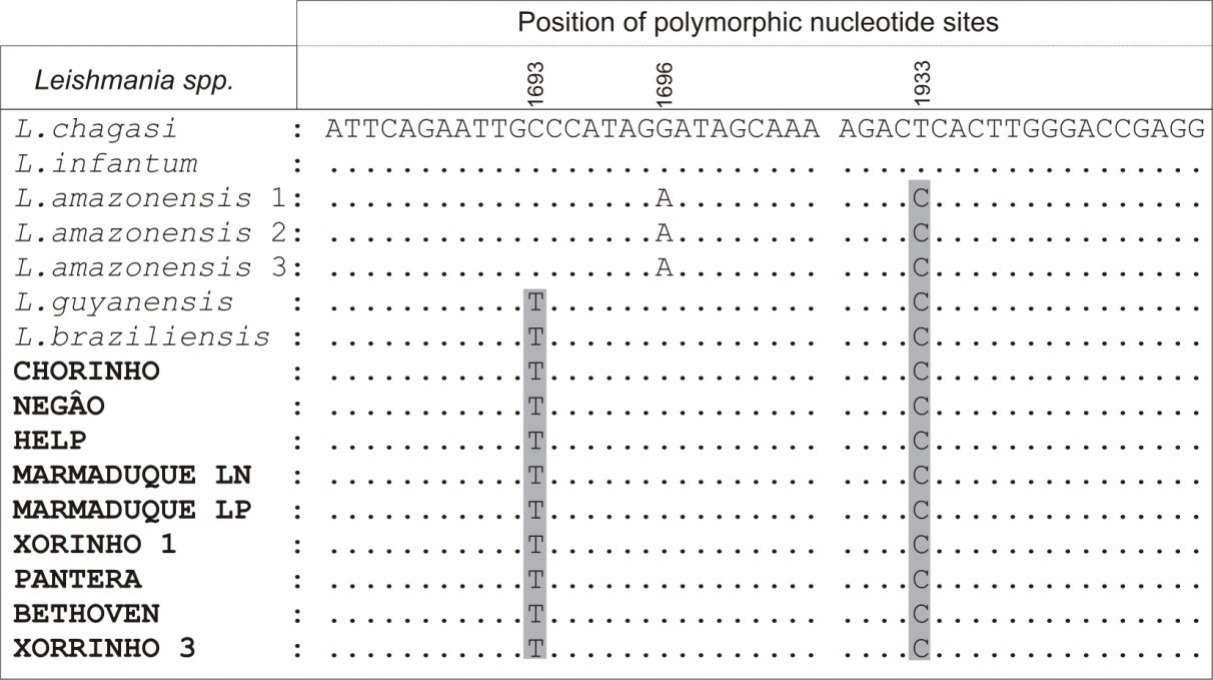
The figure depicts the alignment of nucleotide sequences based on the SSU rRNA gene of isolates which were characterized in this study (in bold), as well as compared to sequences of *Leishmania* species deposited in the GenBank such as *L*. *infantum* (XR001203206), *L*. *chagasi* (KJ697713), *L*. *amazonensis* 1 (JX030083), *L*. *amazonensis* 2 (JX030084), *L*. *amazonensis* 3 (JX030085) *L*. *guyanensis* (KF041803) and *L*. *braziliensis* (JX030135).

All the eight new isolates from dogs showed identical sequences and BLAST analysis demonstrated that they were closest to *Leishmania* spp. of the *Viannia* subgenus. The sequences were aligned using Clustal X and manually refined. Moreover, alignment was created, in the present study, by aligning the SSU rRNA sequences (~436 bp) of the novel samples with those of other species available in the GenBank (Fig 2).

In analysis of ITS1, six biopsies were found to be positive for *Leishmania* spp. Further, an alignment was created with new isolates and the representative species of the subgenus *Viannia*; i.e. *L*. *(V*.*) braziliensis* (type 1 and 2), *L*. *(V*.*) peruviana*, *L*. *(V*.*) guyanensis*, *L*. *(V*.*) naiffi*, and *L*. *(V*.*) lainsoni*. *L*. *(L*.*) amazonensis* and *L*. *(L*.*) mexicana* were also included. Apart from this, the representative sequences of new isolates have been submitted in the GenBank database under accession numbers MH382106; MH382107 and MH382106 (S1 Table).

**Fig 3:**
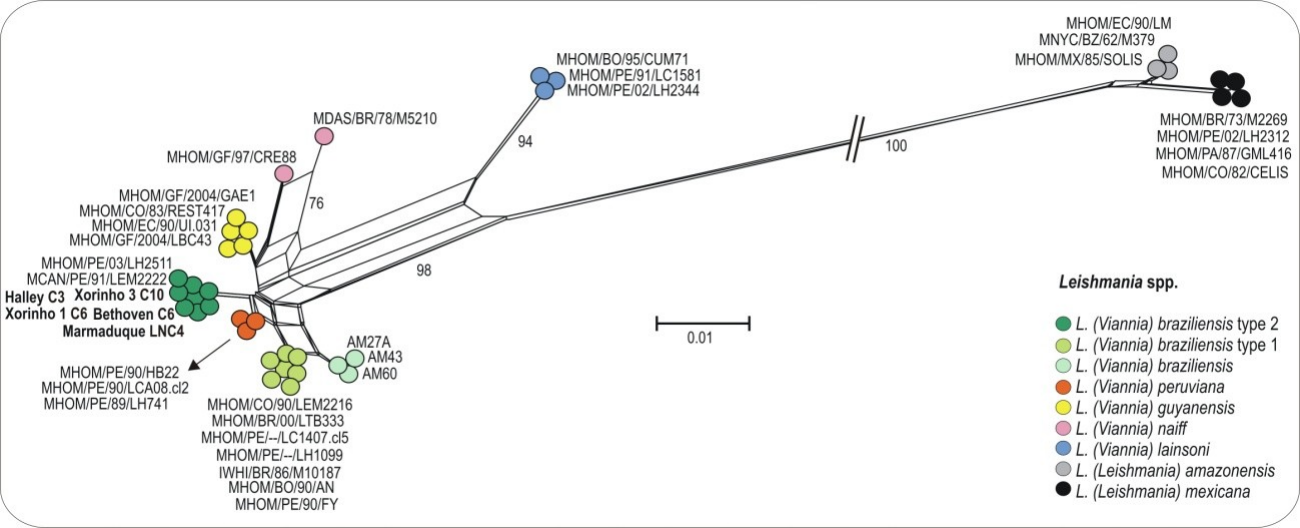
The figure shows the network genealogy using ITS1 rDNA sequences from isolates of *Leishmania braziliensis* (type 1 and 2) and other *Leishmania* spp. Numbers in nodes correspond to support values estimated by 100 bootstrap replicates using the same parameter, which was optimized for network inferences. The isolates characterized in this study are indicated in bold and compared to other isolates of *Leishmania* spp. used in Van der Auwera et al. (2014) and Avila et al. (2018).

On the other hand, the network topology of ITS1 sequences separated all *Leishmania* species from the subgenus *Viannia* and *Leishmania*, and showed that the isolates characterized in this study are identical with or very similar to *L*. *(V*.*) braziliensis* type 2 described in a dog (MCAN / PE / 91 / LEM2222) and man (MHOM / PE / 03 / LH2511) from Peru (Fig 3).

In HRM analysis, 14 samples (12 animals) were examined, of which 11 samples showed positive results in hsp70 real-time PCR (Table 2). The HRM analysis was performed on all the positive samples using three different amplicons, each of them with a different power of discrimination. Further, a difference of 0.25°C was used as a cut-off for a sample to be identified in comparison with the standards.

**Table 2:** The table presents the hsp70 amplicons 1, 2, and 3 of DNA, from each sample, submitted to HRM analysis.

	HRM Identification		
Samples	Amplicon 1	Amplicon 2	Amplicon 3
Dog 01 –Chorinho	GUY	BRA NAI	MEX NAI SHA
Dog 02 –Hulk	negative	negative	negative
Dog 03 –Halley	variant	BRA NAI	MEX NAI SHA
Dog 05 –Help	BRA GUY	BRA NAI	INF BRA GUY
Dog 06 –Negão	variant	BRA NAI	MEX NAI SHA
Dog 11 –Valente	negative	negative	negative
Dog 15 –LN–Xapuri	GUY	BRA NAI	MEX NAI SHA
Dog 15 –LP–Xapuri	negative	negative	negative
Dog 26 –LN–Marmaduque	GUY	BRA NAI	MEX NAI SHA
Dog 26 –LP–Marmaduque	GUY	BRA NAI	MEX NAI SHA
Dog 37 –Xorinho 1	GUY	BRA NAI	MEX NAI SHA
Dog 39 –Pantera	GUY	BRA NAI	MEX NAI SHA
Dog 40 –Bethoven	GUY	BRA NAI	MEX NAI SHA
Dog 41 –Xorinho 3	variant	BRA NAI	MEX NAI SHA

GUY: *L*. *(V*.) *guyanensis*; BRA: *L*. *(V*.) *braziliensis*; NAI: *L*. *(V*.) *naiffi*; SHA: *L*. *(V*.) *shawi*; MEX: *L*. (*L*.) *mexicana*; INF: *L*. (*L*.) *infantum*

**Fig 4:**
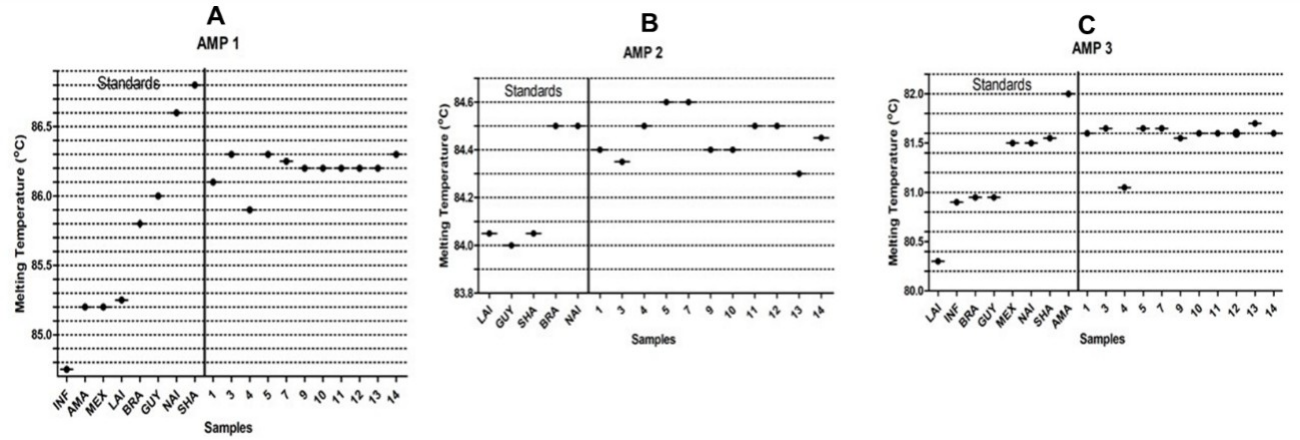
The figure presents the melting temperatures (Tm) for Amplicons 1 (Fig 4A), 2 (Fig 4B), 3 (Fig 4C), and standard species. The plots show the average Tm values. Each species and sample was tested in duplicate. INF: *L*. (*L*.) *infantum*; AMA: *L*. (*L*.) *amazonensis*; MEX: *L*. (*L*.) *mexicana*; LAI: *L*. (*V*.) *lainsoni*; BRA: *L*. (*V*.) *braziliensis*; GUY: *L*. (*V*.) *guyanensis*; NAI: *L*. (*V*.) *naiffi*; SHA: *L*. (*V*.) *shawi*. (1) Dog 01 –Chorinho; (2) Dog 02 –Hulk; (3) Dog 03 –Halley; (4) Dog 05 –Help; (5) Dog 06 –Negão; (6) Dog 11 –Valente; (7) Dog 15 –LN–Xapuri; (8) Dog 15 –LP–Xapuri; (9) Dog 26 –LN–Marmaduque; (10) Dog 26 –LP–Marmaduque; (11) Dog 37 –Xorinho 1; (12) Dog 39 –Pantera; (13) Dog 40 –Bethoven; (14) Dog 41 –Xorinho 3.

Amplicon 1 was able to group the standard species, based on melting temperature values, in four clusters, as shown in Fig 4A. Using this amplicon as a target for identification, seven samples presented the same Tm value as the *L*. (*V*.) *guyanensis* standard; one sample presented Tm value overlapping the *L*. (*V*.) *braziliensis* and *L*. *(V*.) *guyanensis* standards; and three samples presented values distinct from all the standards and were further classified as “variant”. In the Amplicon 2, groups of species from the subgenus *L*. (*Viannia*) were grouped in two clusters, with all the positive samples presenting Tm values similar to the standards of *L*. *(V*.*) braziliensis* and *L*. (*V*.) *naiffi* (Fig 4B). The Amplicon 3 was able to group the standard in four clusters. One sample presented a Tm value similar to that of *L*. *(L*.*) infantum*, *L*. *(V*.*) braziliensis*, and *L*. *(V*.*) guyanensis*, and all other samples presented Tm values similar to the standards of *L*. (*L*.) *mexicana*, *L*. (*V*.) *naiffi*, and *L*. (*V*.) *shawi* (Fig 4C).

The simultaneous analyses of the 3 amplicons, with the results identified, as well as taking into consideration the fact that Amplicon 2 is subgenus *L*. *(Viannia)*-specific, only one sample was recognized, as *L*. (*V*.) *braziliensis* (Dog 05 –Help) while all the other positive samples were identified as “*L*. *(Viannia*) variants” since they all presented intermediate profiles as compared to the standards (Fig 4 and Table 2).

**Fig 5:**
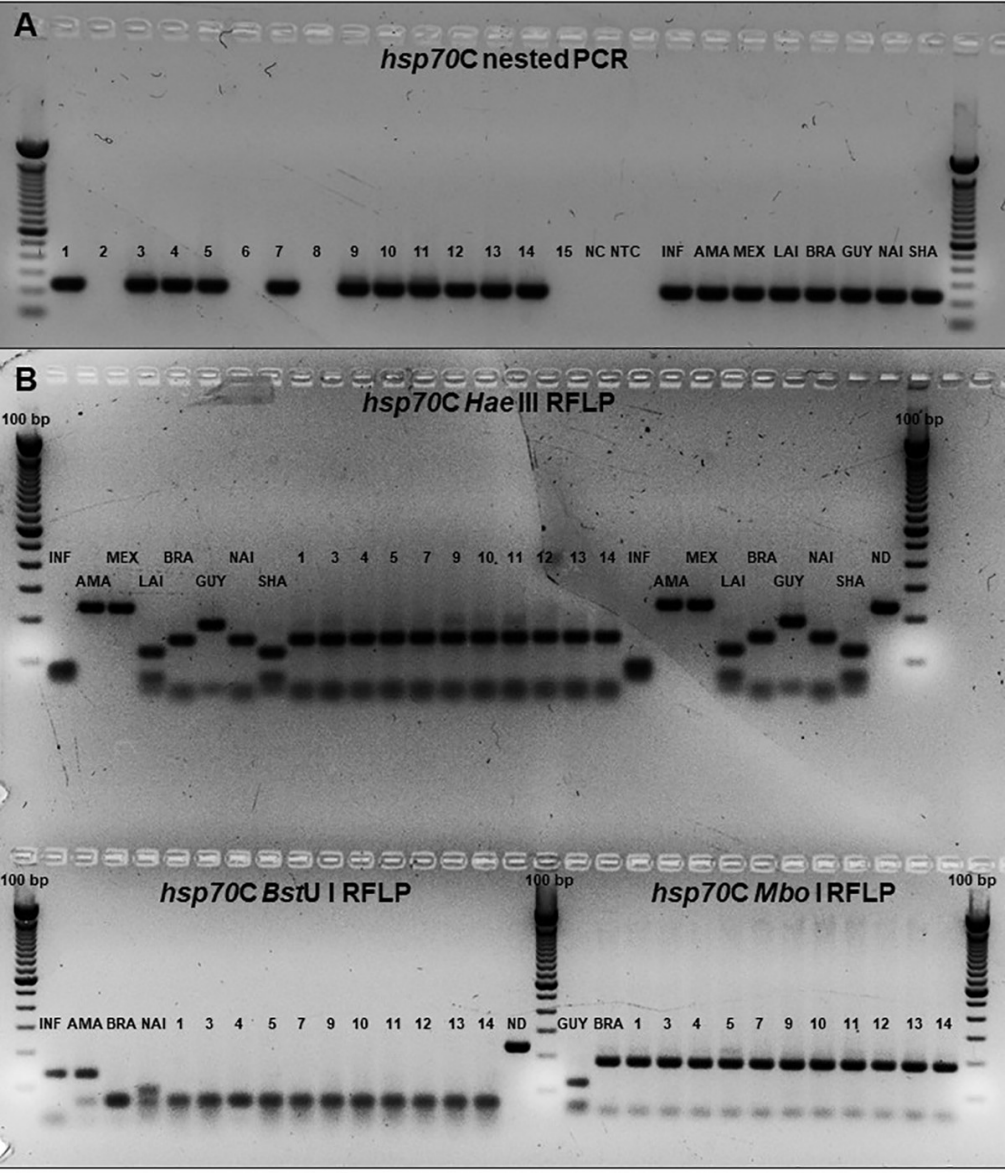
The figure displays the hsp70C nested PCR and hsp70C-RFLP profiles. PCR products (A) and RFLP products (B,) were separated in a 3% agarose gel electrophoresis, stained with ethidium bromide and visualised under UV light. INF: *L*. *(L*.*) infantum;* AMA: *L*. *(L*.*) amazonensis;* MEX: *L*. *(L*.*) mexicana;* LAI: *L*. *(V*.*) lainsoni;* BRA: *L*. *(V*.*) braziliensis;* GUY: *L*. *(V*.*) guyanensis;* NAI: *L*. *(V*.*) naiffi;* SHA: *L*. *(V*.*) shawi*. ND undigested fragment; NC: negative control (NTC from preamplification reaction used as template in the nested PCR); NTC: Non template control. (1) Dog 01 –Chorinho; (2) Dog 02 –Hulk; (3) Dog 03 –Halley; (4) Dog 05 –Help; (5) Dog 06 –Negão; (6) Dog 11 –Valente; (7) Dog 15 –LN–Xapuri; (8) Dog 15 –LP–Xapuri; (9) Dog 26 –LN–Marmaduque; (10) Dog 26 –LP–Marmaduque; (11) Dog 37 –Xorinho 1; (12) Dog 39 –Pantera; (13) Dog 40 –Bethoven; (14) Dog 41 –Xorinho 3.

In PCR-RFLP hsp70C analysis, 11 of 14 tested samples gave positive results in hsp70C nested PCR (Fig 5A). *Hae*III digestions of all hsp70C positive samples produced profiles similar to *L*. *(V*.*) braziliensis* and *L*. *(V*.*) naiffi* standards. Further, as *Mbo*I and *Bst*UI digestions can differentiate these two species, the simultaneous analysis of the three polymorphism profiles was able to classify all the positive samples as *L*. *(V*.*) braziliensis* (Fig 5B).

## Discussion

The *Leishmania braziliensis* complex is the main etiological agent of the dermotropic forms of leishmaniasis in the Americas, with different clinical and epidemiological implications. Currently, this complex comprises two closely related species, (1) *L*. *peruviana* which is limited to the Andean regions, and (2) *L*. *braziliensis*, which is widely distributed in South America, with the highest occurrence in the Amazon region [19–21].

In the present study, a high rate of infection by *Leishmania* with clinical manifestation for ACL in domestic dogs was detected, with the parasite identified being very close to a variety of *L*. (*V*.) *braziliensis*, isolated from human and canine cases from Peru [22], the authors suggest that this parasites are genetically atypical, belonging to a distinct subgroup, further denominated as *L*. (*V*.) *braziliensis* type 2. On a similar note, a previous AFLP analysis of the genome clearly demonstrated that the group was an entity distinct from *L*. *braziliensis*. The authors also report that although the parasites belonging to this group have been isolated from mucosal lesions, the clinical relevance of *L*. *braziliensis* type 2 is not yet recognized nor documented [22–24]. However, the present investigation demonstrates that the lesions found in dogs mostly occur in the cutaneous form, but in some animals, in mucocutaneous form. Therefore, reinforcing the hypothesis that *L*. *braziliensis* type 2 may cause the mucocutaneous clinical form.

Interestingly, the isolates from Peru were from the Department of Huánuco, located at 1800 meters altitude on the eastern slopes of the Andes, and with this new report, it is suggested that *L*. *braziliensis* type 2 is both trans and cis-Andean. Moreover, phlebotomine species from Andean regions have also been found in the Acre state [25], which also reinforces the hypothesis that the Andean region gave rise to American leishmaniasis [26].

The state of Acre has a high diversity of *Leishmania* species causing human cases of ACL [27, 28], and sandflies have been found naturally infected with *L*. *braziliensis* and *L*. *guyanensis* [29, 30]. Upon carrying out studies on natural infection by *Leishmania* of the phlebotomine fauna conducted by Ávila *et al*. [30] in Rio Branco municipality, ITS1 sequences with 99% and 100% identity with *L*. *braziliensis*, were deposited in Genbank, differing from *L*. *braziliensis* type 1 and the *L*. *braziliensis* type 2 found in Xapuri and Peru, revealing that different populations of *L*. *braziliensis* circulate in the state of Acre. Furthermore, several human cases of ACL associated with strains genetically related to *L*. *braziliensis* have been reported in circumscribed areas of Amazonia [31].

The absence of *Leishmania* DNA diagnosed by PCR or flagellate forms in cultures of animal blood samples is in accordance with the observations of some authors who have also used biological samples in the diagnosis of ACL. The low sensitivity of the tests and few circulating parasites may explain these negative results [8, 32, 33] or by the fact that the hematogenic spread of the disease has been controlled by the immune system of these animals [34].

High rates of infection in dogs by *Leishmania*, generally attributed to *L*. *braziliensis*, in areas where human ACL occurs, have been reported in several parts of Brazil [6–8, 35] and also in other Latin American countries [36, 37]. However, some discussion has begun with the major emphasis on elucidating the role of the dog as a possible reservoir of this etiological agent [3, 38, 39]. Apart from this, one also observed that in the study area, the animals live close to the forest, with the presence of reservoirs, in addition to, proven and suspected *Leishmania* vectors. Thus, these populations, both human and canine, are exposed to two cycles of transmission of *Leishmania*, a sylvatic one due to predatory activities, and a peridomestic one, because the residences are close to forest environments and frequented by vectors from the sylvatic cycle, making it possible for these dogs to acquire leishmaniosis, these transmission profiles in dogs have also been reported in other regions of Brazil [35, 40, 41].

The presence of *L*. *braziliensis* type 2 in canine and human cases (unpublished work) is an indication that its occurrence is more widespread out than that of the Peruvian Andes where it was first isolated. Its connectivity with the Amazon Biome was also established. This finding shows the need for further studies on more sensitive methods of diagnosis to detect *L*. *braziliensis* type 2 infection and its accurate identification, in order to know the epidemiological profile of the human and canine population infected by this parasite and also its reservoirs and vectors. Futhermore, new studies regarding the role of domestic dogs in the transmission cycle of ACL etiological agents, as well as their interaction with vectors to better understand their epidemiological involvement is urgently needed.

## Supporting information

S1 Table *Leishmania* spp. and their respective sequences from genes determined in this study (bold) and retrieved from Genebank. (DOCX)
