# Supplementary material for: Leishmania (Viannia) braziliensis type 2 as probable etiological agent of canine cutaneous leishmaniasis in Brazilian Amazon
Source: PLoS One. 2019 Apr 30;14(4):e0216291. doi: 10.1371/journal.pone.0216291 (PMC6490954; doi:10.1371/journal.pone.0216291)
Supplement: S1 Table — (DOCX) [file pone.0216291.s001.docx]

| ***Leishmania spp.***  **Isolates (WHO code)** | **Host Origin** | | **Geographic Origin** | **GenBank accession number** | |
| --- | --- | --- | --- | --- | --- |
|  |  |  |  | **SSU rRNA** | **ITS1 rRNA** |
| ***L. (V.) braziliensis*** **type 2** | |  |  |  |  |
| MHOM/PE/03/LH2511 | human | *Homo sapiens* | Peru |  | HG512966 |
| MCAN/PE/91/LEM2222 | dog | *Canis familiaris* | Peru |  | HG512930 |
| Marmaduque LN | dog | *Canis familiaris* | Acre, Brazil | **MH382106** | **MH382108** |
| Halley | dog | *Canis familiaris* | Acre, Brazil | **MH382106** | **MH382108** |
| Xorinho 1 | dog | *Canis familiaris* | Acre, Brazil | **MH382106** | **MH382107** |
| Bethoven | dog | *Canis familiaris* | Acre, Brazil | **MH382106** | **MH382107** |
| Xorinho 3 | dog | *Canis familiaris* | Acre, Brazil | **MH382106** | **MH382107** |
| Xapuri LN | dog | *Canis familiaris* | Acre, Brazil | **MH382106** | **MH382107** |
| ***L. (V.) braziliensis*** **type 1** | |  |  |  |  |
| MHOM/BO/90/AN | human | *Homo sapiens* | Bolivia |  | HG512938 |
| MHOM/BR/00/LTB333 | human | *Homo sapiens* | Brazil |  | HG512942 |
| MHOM/CO/90/LEM2216 | human | *Homo sapiens* | Colombia |  | HG512929 |
| MHOM/PE/90/FY | human | *Homo sapiens* | Peru |  | HG512937 |
| MHOM/PE/--/LH1099 | human | *Homo sapiens* | Peru |  | HG512901 |
| MHOM/PE/--/LC1407.cl5 | human | *Homo sapiens* | Peru |  | HG512898 |
| IWHI/BR/86/M10187 | phlebotomine | *Lutzomyia whitmani* | Brazil |  | HG512940 |
| AM27A | phlebotomine | *Psychodopygus llanosmartinsi* | Brazil |  | MF802812 |
| AM43 | phlebotomine | *Pintomyia nevesi* | Brazil |  | MF802818 |
| AM60 | phlebotomine | *Psychodopygus ayrozai* | Brazil |  | MF802822 |
| ***L. (V.) naiffi*** |  |  |  |  |  |
| MHOM/GF/97/CRE88 | human | *Homo sapiens* | French Guiana |  | HG512950 |
| MDAS/BR/78/M5210 | armadillo | *Dasypus sp* | Brazil |  | HG512903 |
| ***L. (V.) lainsoni*** |  |  |  |  |  |
| MHOM/PE/02/LH2344 | human | *Homo sapiens* | Peru |  | HG512904 |
| MHOM/BO/95/CUM71 | human | *Homo sapiens* | Bolivia |  | HG512895 |
| MHOM/PE/91/LC1581 | human | *Homo sapiens* | Peru |  | HG512899 |
| ***L. (V.) guyanensis*** |  |  |  |  |  |
| MHOM/CO/83/REST417 | human | *Homo sapiens* | Colombia |  | HG512915 |
| MHOM/GF/79/LEM85 | human | *Homo sapiens* | French Guiana |  | HG512905 |
| MHOM/EC/90/UI.031 | human | *Homo sapiens* | Ecuador |  | HG512935 |
| MHOM/GF/2004/GAE1 | human | *Homo sapiens* | French Guiana |  | HG512960 |
| MHOM/GF/2004/LBC43 | human | *Homo sapiens* | French Guiana |  | HG512961 |
| ***L. (V.) peruviana*** |  |  |  |  |  |
| MHOM/PE/90/HB22 | human | *Homo sapiens* | Peru |  | HG512896 |
| MHOM/PE/90/LCA08.cl2 | human | *Homo sapiens* | Peru |  | HG512900 |
| MHOM/PE/89/LH741 | human | *Homo sapiens* | Peru |  | HG512902 |
| ***L. (L.) amazonensis*** |  |  |  |  |  |
| MHOM/BR/73/M2269 | human | *Homo sapiens* | Brazil |  | HG512964 |
| MHOM/PE/02/LH2312 | human | *Homo sapiens* | Peru |  | HG512965 |
| MHOM/PA/87/GML416 | human | *Homo sapiens* | Panama |  | HG512932 |
| MHOM/CO/82/CELIS | human | *Homo sapiens* | Colombia |  | HG512933 |
| ***L. (L.) mexicana*** |  |  |  |  |  |
| MHOM/EC/90/LM | human | *Homo sapiens* | Ecuador |  | HG512934 |
| MHOM/MX/85/SOLIS | human | *Homo sapiens* | Mexico |  | HG512931 |
| MNYC/BZ/62/M379 | rodent | *Nyctomys sp* | Belize |  | HG512912 |

**S1 Table**. *Leishmania spp.* and their respective sequences from genes determined in this study (bold) and retrieved from Genebank.
